# Evaluation and selection of tandem repeat loci for *Streptococcus pneumoniae *MLVA strain typing

**DOI:** 10.1186/1471-2180-5-66

**Published:** 2005-11-16

**Authors:** Jean-Louis Koeck, Berthe-Marie Njanpop-Lafourcade, Sonia Cade, Emmanuelle Varon, Lassana Sangare, Samina Valjevac, Gilles Vergnaud, Christine Pourcel

**Affiliations:** 1 Laboratoire de biologie clinique, HIA Robert Picqué, 351, route de Toulouse, 33 140 Villenave d'Ornon, France; 2 Association pour l'Aide à la Médecine Préventive, 25-28 rue du Dr Roux, 75 015 Paris, France; 3 Centre National de Référence des Pneumocoques, Laboratoire de Microbiologie, Hôpital Européen Georges Pompidou, 20-40 rue Leblanc, 75908 Paris cedex 15, France; 4 Laboratoire de Virologie-Bactériologie; Centre Hospitalier Universitaire Yalgado Ouédraogo Ouagadougou, Burkina Faso; 5 Génome, Polymorphisme et Minisatellites (GPMS), Institut de Génétique et Microbiologie, Bat. 400, Université Paris XI, 91405 Orsay cedex, France; 6 Division of Analytical Microbiology, Centre d'Etude du Bouchet, BP3, 91710 Vert le Petit, France

## Abstract

**Background:**

Precise identification of bacterial pathogens at the strain level is essential for epidemiological purposes. In *Streptococcus pneumoniae*, the existence of 90 different serotypes makes the typing particularly difficult and requires the use of highly informative tools. Available methods are relatively expensive and cannot be used for large-scale or routine typing of any new isolate. We explore here the potential of MLVA (Multiple Loci VNTR Analysis; VNTR, Variable Number of Tandem Repeats), a method of growing importance in the field of molecular epidemiology, for genotyping of *Streptococcus pneumoniae*.

**Results:**

Available genome sequences were searched for polymorphic tandem repeats. The loci identified were typed across a collection of 56 diverse isolates and including a group of serotype 1 isolates from Africa. Eventually a set of 16 VNTRs was proposed for MLVA-typing of *S. pneumoniae*. These robust markers were sufficient to discriminate 49 genotypes and to aggregate strains on the basis of the serotype and geographical origin, although some exceptions were found. Such exceptions may reflect serotype switching or horizontal transfer of genetic material.

**Conclusion:**

We describe a simple PCR-based MLVA genotyping scheme for *S. pneumoniae *which may prove to be a powerful complement to existing tools for epidemiological studies. Using this technique we uncovered a clonal population of strains, responsible for infections in Burkina Faso. We believe that the proposed MLVA typing scheme can become a standard for epidemiological studies of *S. pneumoniae*.

## Background

*S. pneumoniae *infections remain the major cause of pneumonia, meningitis and otitis in many countries, and a growing number of isolates appear to be resistant to penicillin. Purulent meningitis due to *S. pneumoniae *was recognized more than twenty years ago to be a serious problem in African countries [1] and is known to occur in a seasonal pattern in sub-Saharan Africa [2]. Serotype 1 is one of the most common pneumococcal serotypes associated with disease, although its prevalence varies among countries [3].

Apart from serotyping based on the variations of the coat exopolysaccharides, different DNA-based methods utilize genetic polymorphism. Macrorestriction and Pulsed-Field Gel Electrophoresis analysis (PFGE) [4], and Multiple Loci Sequence Typing (MLST) [5] are the most frequently used genotyping techniques. An MLST typing system was described by Enright *et al*. [5] together with an online identification page. The rep-PCR or BOX PCR assay was described in 1996 by van Belkum [6]. The different techniques have been compared in several studies [7,8]. Other methods use the sequencing of PCR product such as the *gal U *gene [9], or the PCR restriction profile of the *cpsA-cpsB *genes [10].

Although some of these techniques have proven their capacity to discriminate efficiently among the multiple serotypes, the data are not always reproducible between different laboratories, some may not be amenable to the making of international databases, or they are time consuming and expensive. Polymorphic tandem repeat sequences also called Variable Number of Tandem Repeats (VNTR) are an interesting class of genetic markers. Multiple alleles may be present at a single locus, and size differences are easily resolved by electrophoresis of PCR products. Tandem repeat typing has proved to be highly appropriate for the typing of pathogenic bacterial species [11,12], including species with a very high genetic homogeneity such as the *Mycobacterium tuberculosis *complex, *Bacillus anthracis*, and *Yersinia pestis *[13-15].

The availability of genome sequence data from different *S. pneumoniae *strains greatly facilitates the search for polymorphic DNA sequences [16]. In this report, we have evaluated the polymorphism of selected tandem repeats, and measured their discrimination power, across a diverse collection of strains.

## Results

### Selection of VNTRs for MLVA typing

**Figure 1:**
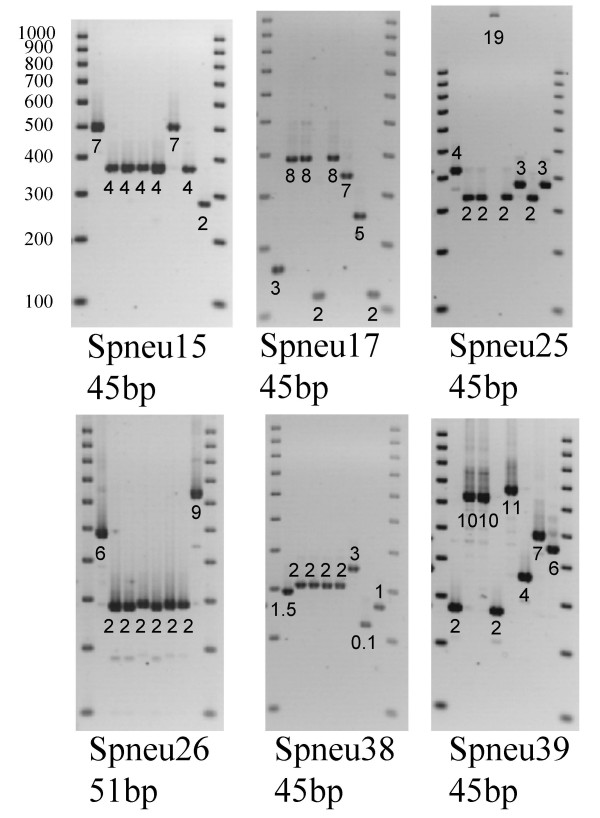
**PCR amplification of 6 VNTRs using DNA of 8 isolates**. From left to right R6, RP31, RP28, RP32, RP29, RP33, RP30 and RP34. A 100-bp ladder (100 to 1000 bp) is run on both side of each group of samples. The number of repeat units within each allele is indicated. The rare alleles of intermediate size are coded "#.5" (for instance Strain R6 is coded 1.5 for locus Spneu38).

At the onset of this study the genome sequences of two strains, R6 and TIGR4 were available. Comparison of these genome sequences using the approach described by Denoeud *et al*. [16,17], identified 33 tandem repeats with a repeat unit equal or larger than 12 bp and predicted to display size polymorphism. Preliminary sequence for two additional genomes (Sanger Spanish 23F-1 and TIGR 670-6B) was subsequently made available and was used to select primers for PCR amplification that would match with all four strains. To confirm that the selected markers were indeed polymorphic, a first set of eight isolates including the reference strain R6 and RP28 to RP34 (Table 1 and Table 2) were analyzed (Fig. 1 and data not shown). Eighteen VNTRs were retained to investigate a larger collection, 12 of which with a 45 bp repeat belong to the BOX family of repeated elements [18] (Table 3). Spneu19, a 60 bp repeats, encodes the choline-binding domain of pneumococcal protein A encoded by *PcpA *[19]. Spneu36, a 45 bp boxB repeat is fused in strain R6 to gene *trzA *encoding the N-ethylammeline chlorohydrolase a Atz/Trz family protein. The sequence diversity within the repeat units of the 18 VNTRs was calculated using the Tandem Repeat Finder software [20], and is indicated as percent matches (Table 3).

### Typing of the reference strain collection

A larger collection of 53 isolates comprising isolates from different origins and with a variety of serotypes was then genotyped (Table 1 and Table 2). As a control, strain R6 was systematically analysed with each set of 5 isolates [15]. The primers listed in Table 3 were used essentially as previously described [14]. The VNTRs were amplified very efficiently in most of the isolates. For a few isolates, no amplification was obtained with Spneu19 and Spneu36. The size variations of the amplicons were as expected for an exact multiple of repeats except in a few cases. With marker Spneu38, an amplicon of intermediate size was observed, marked "1.5", with strain R6 (Figure 1), and for Spneu27 RP36 had a "0.5" intermediate size allele. With marker Spneu25, amplification of RP44 and RP32 (serotype 23F) produced a 1.5 kb amplicon (allele coded "19"). When examining this locus in the different sequenced genomes we found that Sanger strain 23F had an Insertion Sequence (IS) inserted in front of the Spneu25 tandem repeat between the PCR primers. Similarly, for Spneu33, PCR amplification of two isolates, RP43 (serotype 19F) and RP18 (serotype 23F), produced a 2.6 kb amplicon (allele arbitrarily coded "20"), suggesting the presence of an IS element in the repeat. For Spneu 38 and Spneu 42, alleles coded "0.1" correspond to the absence of a VNTR unit although a PCR product is observed.

Spneu26 has a peculiar configuration in strain R6. In this strain 2 repeated elements are observed, a 49 bp repeat with low internal homogeneity inserted inside the usual 51 bp repeat.

**Figure 2:**
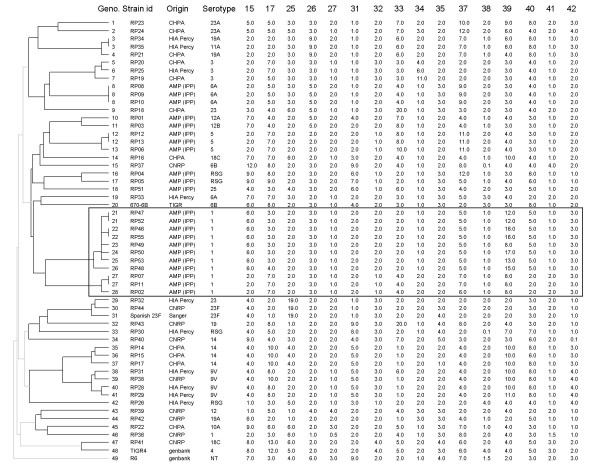
**Dendrogram showing the clustering of 56 isolates by MLVA using 16 markers**. The data are expressed as number of repeats for each of the 16 VNTRs. The genotype number is indicated in the first column "Geno". Some strains possess a rare serogroup (RSG) or were not typable (NT). The serotype 1 isolates from Burkina Faso are boxed.

The putative MLVA profiles of 4 fully or partially sequenced genomes were determined and used in the clustering analysis shown in Figure 2. The data for Spneu19 and Spneu36 were not used for this clustering because of the existence of null alleles in some isolates as mentioned. Combining the 52 isolates of this study (not including RP45 alias R6, represented by the sequenced genome) plus the 4 sequenced strains, 49 genotypes are observed. The global Hunter Gaston diversity index (HGDI) for the described MLVA assay is 0.995. The HGDI for each VNTR marker is shown in Table 3.

The remarkable similarity between independent isolates with the same serotype strongly suggests that these markers, with the exception of Spneu 39, do not vary at a high frequency. This is confirmed by the fact that the size of the 18 VNTR alleles was the same in two independent R6 isolates, and corresponded exactly to those of the sequenced genome.

To assess the reproducibility of the assay, a series of 10 isolates, RP46 to RP55, were genotyped in duplicate, in Orsay and Bordeaux, giving the same fingerprint. The allele size assignment was performed by eye in one site and using the BioNumerics tools in the other site.

### Analysis of two small epidemic groups

Part of the isolates in this study correspond to an epidemic situation. They were isolated in the same geographic area in Burkina Faso during outbreaks of meningitis in year 2002–2003 and 2004. In 2002–2003, 8 out of 9 isolates were of serotype 1 and one was of serotype 25F. The eight serotype 1 isolates from 2002–2003 have identical alleles at all markers except Spneu39. Interestingly, for this marker 6 different alleles are observed, all corresponding to a large number of repeats (8 to 16). In 2004, isolates of 4 different serotypes were obtained, three serotype 6, three serotype 1, three serotype 5 and two serotype 12 isolates. The serotype of RP04 and RP05 could not be determined. All these isolates cluster according to their serotype. The three serotype 1 isolates are identical except for marker Spneu37. Although clearly distinct, they are grouped with the serotype 1 strains isolated in 2002–2003 (shown in a box on Fig. 2).

## Discussion

A collection of 18 VNTR markers which can be used to genotype *S. pneumoniae *strains by simple PCR and agarose gel electrophoresis has been identified. Two of these markers, Spneu19 and Spneu36, belonging respectively to *pcpA *and *trzA*, were not kept in the clustering analysis because they were absent from some isolates, although they might be useful in specific situations. Spneu19 is not amplified in isolates of serotype 3 and 6A suggesting that they lack *pcpA*. It was proposed that the protein encoded by *pcpA *could be a surface protein involved in cell adhesion with specific proteins of the human extracellular matrix. PCPA is not essential for bacterial growth at least under laboratory conditions as the gene can be knocked-out with no noticeable change in the pneumococcal phenotype [19]. However the polymorphism observed at the choline binding domain might play a role during infection. The effect of Spneu36 polymorphism on *trzA *is not clear as the 45 bp repeat is apparently not fused to this gene in the TIGR4 genome.

Among the 18 selected markers, 16 consist in 45 to 60 bp repeats with a regular variation and which amplify very efficiently. The size polymorphism can be scored by eye. Two additional markers of 12 bp and 14 bp repeats are also described. In the present study, VNTRs with smaller repeat units, of the microsatellite category (1 to 8 bp long) were not investigated. Due to their sometimes relatively high mutation rate, they may improve the MLVA resolution to investigate local outbreaks.

Most of the VNTRs correspond to the intergenic 45 bp boxB repeat. They belong to a family of elements, present in multiple loci in the *S. pneumoniae *genome, and composed of three subunits boxA, boxB and boxC. Subunit boxB, 45 bp long, can be tandemly repeated with a high internal sequence similarity [18]. These sequences have been suggested to be regulatory elements shared by coordinately regulated genes. The subunit boxB is the only one that can be tandemly repeated.

The polymorphism of these elements has been used to genotype strains in the BOX-PCR assay. However this assay produces an image (a multi-band pattern) of the added polymorphism of multiple BOX elements whereas the VNTR assay analyses each locus separately. As a result, the MLVA assay is more informative and reproducible, data interpretation is much easier, and genotyping databases can be easily produced [21].

We performed an MLVA analysis on a collection of isolates originating from Africa and France. Clustering was observed both on the basis of serotype and geographical origin although there are some exceptions. Analysis of the sequenced genomes have shown the existence of many gene transfer events which could explain why strains with the same serotype do not always cluster. Conversely, MLVA aggregates a number of strains of serotypes which are known to be close variants, such as serotype 19F and 23F [22], and serotype 14 and 9V [23].

All the African serotype 1 isolates are grouped (Figure 2). Interestingly a cluster of 3 isolates RP07, RP11 and RP02 isolated in 2004 and differing by only one marker, are linked to a second cluster of 9 isolates recovered in 2002–2003 from which they differ at 9 out of 16 markers. This is a large distance suggesting that the existing population of strains able to cause outbreaks is very diverse. A second lineage contains serotype 12 and serotype 5 strains differing at 7 out of 16 markers.

Serotype 1 strains seem to have a propensity to cause meningitis in Burkina Faso, as two related clones of this serotype were found in 50% of the analyzed cases. A similar observation was made in Northern Ghana [24]. In contrast to the majority of other serotypes, nasopharyngeal carriage of serotype 1 is exceptionally observed. This could be linked to 1) a lesser genetic diversity as compared to other serotypes, due to limited exchanges with other streptococci during colonization, 2) a high invasive potential or 3) high attack rates, as supported by the observations of Leimkugel et *al*. [24]. For serotypes other than serotype 1, distribution across the dendrogram may be associated with the nasopharynx carriage (e.g. 23F).

## Conclusion

This preliminary investigation validates a first set of markers for MLVA investigation. The lethality linked to pneumococcal meningitis is high and an appropriate vaccination is necessary, requiring the identification of virulent lineages. The usefulness of the MLVA typing scheme proposed here can now be further determined by investigating a larger population of isolates from Africa which are currently being collected.

However in a species with 90 different genotypes, additional studies will clearly be needed. In particular, it will be useful to see how MLVA compares with MLST [25]. In contrast with MLST, the relatively low cost and moderate expertise required for MLVA typing would allow the systematic typing of any new isolate directly by clinical laboratories within hospitals. All markers proposed here are easy to type with no sophisticated equipment and software, so that it should in principle be feasible to organize networks of clinical laboratories, each one taking in charge the typing of local isolates. To facilitate such projects, shared internet resources enabling the import and analysis of results could be also set-up [21]. Eventually comparison of isolates on such a large scale will provide a precise measure of the stability of each marker, necessary for the optimized interpretation of MLVA typing data.

## Methods

### Bacterial strains

A total of 53 isolates were analyzed (Table 1 and Table 2). RP14 to RP24 and RP25 to RP35 were generous gifts from respectively Hubert Chardon (CHPA, Aix en Provence, France) and Christine Grandpré (Hôpital d'Instruction des Armées, HIA Percy, France). These isolates were obtained from blood or respiratory specimens (i.e. sputums or bronchoalveolar fluids), from patients with severe pneumonia or septicemia or both. RP36 to RP44 are reference strains for serotypes 1, 6B, 9V, 12F, 14, 18C, 19A, 19F and 23F from the Satens Serum Institute (SSI Denmark) and provided to us by the "Centre National de Référence des Pneumocoques" (CNRP France). RP1 to RP13 and RP46 to RP55 were isolated from cerebro-spinal fluid of patients with meningitis in Bobo-Dioulasso (Burkina Faso) by the "Association pour l'Aide à la Médecine Préventive" (AMP IPP) [2]. Strain R6 (ATCC BAA 255) was used as control under the name RP45. The strain was obtained from two sources, one from the CNRP originating from the SSI in Denmark, and the second from the Institut Pasteur collection (CIP 105880). DNA was purified using the InstaGene kit (Biorad, Marnes la Coquette, France). The minimum inhibitory concentrations of antibiotics (MICs) were determined by the E-test (AB Biodisk). Interpretive criteria for susceptibility or resistance were as recommended by the CASFM (Comité de l'Antibiogramme de la Société Française de Microbiologie) [26] (Last release, January 2005).

### Identification of variable number tandem repeats by genomic sequence comparison

The methods previously described [13,16,17,27] were used to identify tandem repeats with a predicted size which differs between the published genome of *S. pneumoniae *strain R6 [28], strain TIGR4 [29] and the preliminary genome 23F (Sanger Institute) and 670-6B (TIGR) sequence data (obtained respectively from [30] and from [31]).

The different tandem repeat loci are designated by using the nomenclature described previously [14]. For instance Spneu1579_45bp_507bp_7u (Spneu15) is a tandem repeat locus at position 1579 Kb in the R6 genome. It has a 45 bp motif, a total PCR product length of 506 bp in the R6 strain when using the primer set indicated in Table 3. This allele size corresponds to 7 units. Its common laboratory name is Spneu15 (Table 3).

### PCR (Polymerase Chain Reaction) amplification and genotyping

PCR amplifications were performed in a total volume of 15 microliters containing 10 ng of DNA, 1× PCR Reaction Buffer, 1U of *Taq *DNA polymerase (Qbiogen, Illkirch, France), 200 microM of each deoxynucleotide triphosphate, and 0.3 microM of each flanking primer. The primers are listed in Table 3. Amplifications were performed in a MJ Research PTC200 thermocycler. Initial denaturation step at 94°C for 5 min. was followed by 30 cycles of denaturation at 94°C for 30 s, primer annealing at 60°C for 30 s, and elongation at 72°C for 45 s. The final extension step was at 72°C for 7 min. Three microliters of amplification product were loaded on a 2% standard agarose gel and run until the bromophenol blue had reached the 20 cm position. Gels were stained with ethidium bromide, visualized under UV light, and photographed (Vilber-Lourmat, Marnes-la-Vallée, France). The size markers used were a 100-bp ladder (EZ Load 100 pb PCR Molecular Ruler, Biorad, Marnes la Coquette, France) or 20-bp ladder (EZ Load 20 pb Molecular Ruler, Biorad, Marnes la Coquette, France) according to the tandem repeat unit length. Gel images were managed using the BioNumerics software package (version 4.0, Applied-Maths, Sint-Martens-Latem, Belgium).

### Data analysis

Band size estimates were converted to number of units within a character dataset. The VNTR data deduced from the sequenced strains R6, TIGR4, 670-6B and Sanger Spanish 23F-1 were added to the MLVA analysis. Clustering analyses used the categorical coefficient and UPGMA (Unweighted Pair Group Method using Arithmetic averages). The use of the categorical parameter implies that the character states are considered unordered. The same weight is given to a large or a small number of differences in the number of repeats at each locus. The polymorphism indexes of individual or grouped VNTRs was calculated using the Hunter-Gaston discriminatory index (HGDI) [32].

## List of abbreviations

VNTR; Variable Number of Tandem Repeats

MLVA; Multiple locus VNTR

HGDI; Hunter Gaston discriminatory index

## Authors' contributions

SC and SV did most of the typing work and CP and GV did the error checking analysis. JLK was in charge of the definition of the study collection and preparation of the DNA samples. BMN, EV and KS collected and provided bacterial isolates. CP initiated and managed the project. GV was in charge of the BioNumerics database and clustering analyses. CP, JLK, and GV wrote the report. All authors read and approved the final manuscript.

## Figures and Tables

**Table 1:** *S. pneumoniae *strains origin and characteristics

Strain id	Reference	Origin	Serotype	Year	Site^a^	Country	Peni G^b^	Ery
RP01	100277	AMP (IPP)	12A	2004	CSF	Burkina Faso	S	S
RP02	200801	AMP (IPP)	1	2004	CSF	Burkina Faso	S	S
RP03	300141	AMP (IPP)	12B	2004	CSF	Burkina Faso	S	S
RP04	400202	AMP (IPP)	RSGa	2004	CSF	Burkina Faso	S	S
RP05	500205	AMP (IPP)	RSG	2004	CSF	Burkina Faso	S	S
RP06	600003	AMP (IPP)	5	2004	CSF	Burkina Faso	S	S
RP07	700025	AMP (IPP)	1	2004	CSF	Burkina Faso	S	S
RP08	800830	AMP (IPP)	6A	2004	CSF	Burkina Faso	S	S
RP09	900518	AMP (IPP)	6A	2004	CSF	Burkina Faso	S	S
RP10	1000244	AMP (IPP)	6A	2004	CSF	Burkina Faso	S	S
RP11	1100236	AMP (IPP)	1	2004	CSF	Burkina Faso	S	S
RP12	1200327	AMP (IPP)	5	2004	CSF	Burkina Faso	S	S
RP13	1300373	AMP (IPP)	5	2004	CSF	Burkina Faso	S	S
RP14	20020876	CHPA	14	2002	Blood	France (sud-est)	R	R
RP15	20020953	CHPA	14	2002	Blood	France (sud-est)	I	S
RP16	20020978	CHPA	18C	2002	Blood	France (sud-est)	S	S
RP17	20021029	CHPA	14	2002	Blood	France (sud-est)	R	R
RP18	20021131	CHPA	23	2002	Blood	France (sud-est)	I	R
RP19	20022003	CHPA	3	2002	Blood	France (south-east)	S	S
RP20	20022042	CHPA	3	2002	Blood	France (south-east)	S	S
RP21	20022215	CHPA	19A	2002	Blood	France (south-east)	I	R
RP22	20022337	CHPA	10A	2002	Blood	France (south-east)	S	S
RP23	20022338	CHPA	23A	2002	Blood	France (south-east)	I	S
RP24	20022339	CHPA	23A	2002	Blood	France (south-east)	S	S
RP25	4030143494	HIA Percy	3	2004	Pulmonary	France (Paris area)	S	S
RP26	488015780	HIA Percy	RSG^c^	2004	Pulmonary	France (Paris area)	S	S
RP27	488019294	HIA Percy	NT^d^	2004	Pulmonary	France (Paris area)	I	R
RP28	4040145233	HIA Percy	9V	2004	Pulmonary	France (Paris area)	I	R
RP29	4050149284	HIA Percy	9V	2004	Pulmonary	France (Paris area)	R	R
RP30	4060150356	HIA Percy	RSG	2004	Pulmonary	France (Paris area)	S	S
RP31	4060150868	HIA Percy	9V	2004	Pulmonary	France (Paris area)	I	R
RP32	4060151258	HIA Percy	23	2004	Pulmonary	France (Paris area)	I	R
RP33	488034644	HIA Percy	6A	2004	Pulmonary	France (Paris area)	S	S
RP34	488037640	HIA Percy	19A	2004	Pulmonary	France (Paris area)	I	S
RP35	4070153283	HIA Percy	11A	2004	Blood	France (Paris area)	S	S
RP46	PED012	AMP (IPP)	1	2002	CSF	Burkina Faso	I	S
RP47	MED042	AMP (IPP)	1	2002	CSF	Burkina Faso	I	S
RP48	PED081	AMP (IPP)	1	2003	CSF	Burkina Faso	S	UN^e^
RP49	MED142	AMP (IPP)	1	2003	CSF	Burkina Faso	S	S
RP50	PED159	AMP (IPP)	1	2003	CSF	Burkina Faso	S	S
RP51	Q06003	AMP (IPP)	25	2003	CSF	Burkina Faso	S	S
RP52	MED126	AMP (IPP)	1	2003	CSF	Burkina Faso	S	S
RP53	Q14004	AMP (IPP)	1	2003	CSF	Burkina Faso	S	S
RP54	PED124	AMP (IPP)	1	2003	CSF	Burkina Faso	S	S
RP55	H01368	AMP (IPP)	1	2003	CSF	Burkina Faso	S	S

(a) Origin of isolate; CSF: Cerebrospinal fluid

(b) Resistance to benzylpenicillin (Pen) or erythromycin (Ery) (S: susceptible; I: intermediate; R: resistant).

(c) RSG rare serogroup

(d) NT non typable

(e) UN unknown

**Table 2:** *S. pneumoniae *reference strains

strain id	Serotype	SSI reference	CNRP Reference
RP36	1	134/95	1172
RP37	6B	105/95	1168
RP38	9V	162/95	1158
RP39	12 F	133/95	1171
RP40	14	109/95	1167
RP41	18C	Holland 1821	1170
RP42	19A	Verbiest 91	1149
RP43	19 F	Verbiest 38	1153
RP44	23 F	Verbiest 51	1156
RP45	NT	R6	105880

**Table 3:** Characteristics of VNTR markers and sequence of the PCR primers

Name	R6 position^a^	Locus	Motif bp	Percent matches	R6 amplicon		PCR primers	No of alleles	Allele size range (units)	HGDI^d ^(56 strains)
					size bp	repeats				
Spneu15	1579	BOX	45	94	507	7	TCCAACACGACCTTTATCCA	10	1–12	0.827
							AGCCTTGCTCCTCATCTTGA			
Spneu17	1783	BOX	45	86	167	3	TCGAAAATCTCTGCAAACCA	12	1–13	0.883
							CGGACTAGGCGGCTGATTA			
Spneu19	1925	*pcpA*	60	98	663	10	TCGGGTGTAGTCGTGTTTACT	5	7–10	0.749
							AACTGATGTAGCTAAACCTAAAAAGAA			
Spneu25	101	BOX	45	96	423	4	TCGCCTTTGCTAATCAAACA	7	1–6^c^	0.744
							GACTGGGTAACAATTCCATT			
Spneu26	185	intergenic	51	65	492	6	ATGGAACAGAAGGCGAATTG	7	1–9	0.688
							AACAAGGCCAGGATTTTCGT			
Spneu27	257	BOX	45	95	347	3	TCAGGAACAGCTATTATCCC	5	0.5–4	0.575
							CCAACCTCCTTTTCGTTTCA			
Spneu31	557	BOX	45	98	594	9	CTGGAATAGTCCATCGAGCTT	9	1–9	0.763
							CGAAGGTAAATGTGAACAAAC			
Spneu32	571	BOX	45	86	280	2	AAAGCAAAATAATGCGCTCCT	4	1–4	0.629
							ACTACCAAAGCCCAAGTGGT			
Spneu33	698	BOX	45	95	407	2	CAGCTGAACATGATGGCAAA	10	1–10^c^	0.858
							CATCACTTCTCCCTTCTAATC			
Spneu34	1082	BOX	45	97	239	1	CTCGGTAAAGACGAGGTTCAA	6	1–11	0.598
							CATCGTGCAGGGATTTTCTAA			
Spneu35	1166	intergenic	49	100	349	4	ACAATCTCAGCTACGCCCTA	5	1–5	0.561
							ACGGGATGACATTAAAAACC			
Spneu36	1209	*trzA*	45	86	320	2	GAAATCTTGATCACAAGTCAC	10	1–10	0.866
							AAAAAGTTGCCTTCGAGTGAC			
Spneu37	1350	BOX	45	91	501	7	ATGCGCAAATCGATTAAGGA	11	2–12	0.876
							CGATGTGCTTCACTCACTCC			
Spneu38	1911	BOX	45	84	297	1.5^b^	TCAGGAGTAGGTTCCTGACCA	6	0.1–4	0.616
							AACCCGAGAAGAAGTGCAAG			
Spneu39	1915	BOX	45	83	275	2	CCTTGGACTACCACCTCGTT	14	2–17	0.862
							GCCGTGACAGACTTCTGGAA			
Spneu40	1611	BOX	45	88	376	3	AGTAGTCTGCAATCGCAGCT	7	2–8	0.832
							GGGCCTTAAGTCGCTTTGAT			
Spneu41	394	intergenic	14	100	166	2	ACCGTAATGGGACTTCATCT	5	1–4	0.484
							ATCTGCACCTAAGACAATCG			
Spneu42	1036	intergenic	12	91	120	3	TGCTCCCTCTGAAAAGTCAT	6	0.1–4	0.739
							CTTTATATTATATCATGCCTTC			

^a ^Position on the chromosome expressed in kbp

^b ^Expected size for a truly 2U amplicon is 312 bp long

^c ^Additional large alleles probably contain an IS element

^d ^Hunter Gaston Diversity index
